# Cancer-selective, single agent chemoradiosensitising gold nanoparticles

**DOI:** 10.1371/journal.pone.0181103

**Published:** 2017-07-10

**Authors:** Sophie Grellet, Konstantina Tzelepi, Meike Roskamp, Phil Williams, Aquila Sharif, Richard Slade-Carter, Peter Goldie, Nicky Whilde, Małgorzata A. Śmiałek, Nigel J. Mason, Jon P. Golding

**Affiliations:** 1 School of Life, Health & Chemical Sciences, The Open University, Walton Hall, Milton Keynes, United Kingdom; 2 Midatech Pharma, Milton Park, Abingdon, United Kingdom; 3 GenesisCare, Milton Keynes Medical Centre, Milton Keynes, United Kingdom; 4 Radiotherapy Department, Northampton General Hospital NHS Trust, Northampton, United Kingdom; 5 Department of Control and Power Engineering, Faculty of Ocean Engineering and Ship Technology, Gdansk University of Technology, Gdansk, Poland; 6 School of Physical Sciences, The Open University, Walton Hall, Milton Keynes, United Kingdom; National Cheng Kung University, TAIWAN

## Abstract

Two nanometre gold nanoparticles (AuNPs), bearing sugar moieties and/or thiol-polyethylene glycol-amine (PEG-amine), were synthesised and evaluated for their *in vitro* toxicity and ability to radiosensitise cells with 220 kV and 6 MV X-rays, using four cell lines representing normal and cancerous skin and breast tissues. Acute 3 h exposure of cells to AuNPs, bearing PEG-amine only or a 50:50 ratio of alpha-galactose derivative and PEG-amine resulted in selective uptake and toxicity towards cancer cells at unprecedentedly low nanomolar concentrations. Chemotoxicity was prevented by co-administration of N-acetyl cysteine antioxidant, or partially prevented by the caspase inhibitor Z-VAD-FMK. In addition to their intrinsic cancer-selective chemotoxicity, these AuNPs acted as radiosensitisers in combination with 220 kV or 6 MV X-rays. The ability of AuNPs bearing simple ligands to act as cancer-selective chemoradiosensitisers at low concentrations is a novel discovery that holds great promise in developing low-cost cancer nanotherapeutics.

## Introduction

Radiotherapy is currently used in around half of all cancer treatments. Although generally effective, it is damaging to surrounding healthy tissues and needs to be improved by better targeting of cancer cells. One promising approach is to use nanoparticles composed of high atomic number elements, such as gold, hafnium, gadolinium, platinum or iron, which have large X-ray photon capture cross-sections, and can therefore locally increase the energy deposition near the nanoparticle through secondary electron emission from the nanoparticles [1–3]. Because of their biocompatibility and amenability to surface modification for tumour targeting, gold nanoparticles (AuNPs) have predominantly been used for tumour radiosensitisation studies [4–6]. AuNP radiosensitisation with external beam sources is more effective when using kilovoltage X-ray photons [7] than with megavoltage X-ray photons [4,8–10], although megavoltage is preferable due to its deeper tissue penetration.

The toxicity of AuNPs depends on their ligand shell, but in general they are non-toxic, except at high concentrations where they generate appreciable levels of reactive oxygen species (ROS) [11,12]. AuNPs smaller than 6 nm hydrodynamic diameter are preferable for therapeutic applications, since these can be excreted from the body by renal clearance, reducing long-term exposure to other organs [13,14].

Chemoradiosensitisers are dual-action drugs that are directly toxic to cells and also render the DNA more susceptible to radiation-induced damage. They include inhibitors of topoisomerase I, poly ADP-ribose polymerase (PARP), histone deacetylase (HDAC) and heat-shock protein 90 (Hsp90) [15]. However, current chemoradiosensitisers lack the ability to locally increase the deposited dose of radiation within cells. With that goal in mind, we have designed novel 2 nm gold core nanoparticles, coated with sugar ligands to improve aqueous solubility [16], and PEG-amine to improve biocompatibility [17] and cellular uptake [18]. Although originally envisaged as radiosensitising platforms to co-deliver anti-cancer drugs, these novel AuNPs were found to be selectively toxic for cancer cells at nanomolar concentrations and also act as radiosensitisers.

## Materials and methods

### Nanoparticle synthesis

Gold nanoparticles (AuNPs) with a mean gold core diameter of 2 nm were prepared by Midatech Ltd (Abingdon, UK) with different input ratios of HS-C_2_-sugar (α-galactose derivative, β-glucose derivative, or N-acetyl glucosamine derivative) and 1-amino-6-mercapto-hexaethyleneglycol (PEG-amine), as described previously [19]. Colloidal, citrate-capped AuNPs with a mean diameter of 2 nm were purchased from BBI Solutions (Cardiff, UK) and used with no further modification. All AuNP concentrations are quoted on the basis of Au content.

### Nanoparticle physical characterisation

#### Transmission electron microscopy (TEM)

Nanoparticles were characterised by TEM imaging on electrostatically discharged carbon grids, using a JEM-1400 microscope (JEOL, USA) at an accelerating voltage of 80 kV and a magnification of x200,000. Nanoparticle size was measured from thresholded TEM images using ImageJ software.

#### Zeta potential

The charge of the nanoparticles (500 μg/ml) was measured in 3.2% PBS pH 7.4 using a zetasizer (Nano ZSP, Malvern instruments, using a DTS1070 cell).

#### Dynamic light scattering

The hydrodynamic size of the nanoparticles (100–400 μg/ml) was measured in water using a zetasizer (Nano ZSP, Malvern Instruments, using a DTS1070 cell).

#### ^1^H-NMR

Three batches of nanoparticles with different input ratios of α-Gal and PEG-amine (25:75, 50:50, 75:25) were synthesised using 10 mg of Au and 3-fold molar excess of ligands.

Four mg of each AuNP were concentrated on 10 kDa cut-off ultrafiltration columns (Amicon) and then washed 3 times with 2 ml of D_2_O. AuNP samples were then transferred to a vial and resuspended in 400μl D_2_O containing 0.3 M KCN and 0.1 M KOH and incubated at 37.5°C overnight. Samples were then centrifuged at 13,000 x *g* for 1 min and analysed by ^1^H-NMR at 500 MHz (Avance III HD, Bruker), using MestReNova software. The defining protons for the α-Gal and PEG-amine ligands were identified to resonate at 4.95 ppm and 2.75 ppm respectively, these correspond to the single anomeric proton of α-galactose (NMR doublet) and the two CH_2_ protons proximal to the terminal NH_2_ bond in the PEG-amine linker (NMR triplet).

### Cell culture

Four cell lines were used, representing normal and cancerous skin cells (HaCaT and HSC-3, respectively) as well as normal and cancerous breast cells (MCF-10 and MCF-7, respectively). HaCaT cells were a gift of Erik Walbeehm, Erasmus Medical Centre, Rotterdam. MCF-7 cells were a gift of Marilena Loizidou, University College London. MCF-10 cells were a gift of Kevin Prise, Queens University Belfast. HSC-3 cells were purchased from the American Type Culture Collection.

HSC-3, HaCaT and MCF-7 cells were grown in low glucose DMEM, supplemented with 10% FCS and 1% penicillin/streptomycin. MCF-10 cells were grown in DMEM/F12 without phenol red, supplemented with: 5% horse serum, 2.5 mM L-glutamine, 15 mM HEPES, 1 ng/ml cholera toxin, 10 μg/ml insulin, 50 μM hydrocortisone, 100 μM EGF and 1% pen/strep. All cells were maintained in T-75 culture flasks and passaged at 80% confluency.

### Clonogenic assay

Cell proliferation and survival were quantified by clonogenic assay. Cells were seeded at between 300–2000 per well in 24 well culture plates and allowed to adhere for 24 h. Nanoparticles were added to the medium for 1, 3, 6 or 24 h, and then washed off once with medium. Cells were cultured for up to 6 days, changing medium every 2–3 days. Cells were then washed and stained with 10 mg/ml methylene blue in 50% ethanol. Colonies containing 50 cells or more were counted and colony formation expressed as a percentage of untreated controls. In some experiments, HSC-3 and HaCaT cells (3600 cells/ml) were loaded with AuNPs for 3 h in suspension culture in 15 ml Falcon tubes, resuspending the cells every 30 min. Cells were then washed twice by centrifugation and seeded at 300 cells per well for clonogenic assay, as described. The IC50 is defined as the concentration of AuNP resulting in 50% reduction in the number of cell colonies, compared with untreated controls. IC50 values were calculated from plots of the logarithm of AuNP concentration versus the percentage cell colonies, using GraphPad Prism 6.0.

### Cellular uptake

Cellular uptake of AuNPs was assessed by TEM for sub-cellular localization, and quantitatively by inductively-coupled plasma mass spectrometry (ICP-MS).

#### Transmission electron microscopy (TEM)

HSC-3 and HaCaT cells were seeded onto 12 well transwell inserts overnight and then incubated for 3 h with 10 μg/ml AuNPs. This AuNP concentration was chosen in initial experiments, as it was the lowest concentration that gave good light microscopy staining of AuNPs in fixed cells, using a silver stain enhancement kit (R-Gent, Aurion). The cells were then fixed and silver enhanced to stain AuNPs and processed for TEM according to Gromnicova *et al* [20]. Ultrathin sections were analyzed using a JEM-1400 microscope with an accelerating voltage of 80 kV at magnifications of x2500 and x20,000.

#### Inductively coupled plasma mass spectrometry (ICP-MS)

HSC-3 and HaCaT cells (500,000 /ml) were incubated for 3 h with AuNPs at their HSC-3 IC50 concentration in suspension culture, resuspending the cells every 30 mins by gentle shaking. Cells were then washed twice with 5 ml medium by centrifugation and the cell density measured by haemocytometer. The cell pellet was dissolved in 2.5 ml of 3% tetramethylammonium hydroxide and 0.2% Triton X-100. Then, 2.5 ml of 1% HCl with iridium as internal standard was added prior to the ICP-MS analysis. The gold amount was calculated against a calibration curve ranging from 0.1 to 100 ng/ml of gold, including a blank (zero) point. Gold determination was performed using a Perkin-Elmer NexION 300X ICP-MS with a NexION ICP-MS software version 1.4.

### Antioxidant cell protection assay

Adherent HSC-3 cells at 300 cell per well were incubated with AuNPs at their IC50 concentration for 3 h, with or without either 1 mM N-acetylcysteine or 0.1 mM sodium pyruvate antioxidants. Cells were washed and fresh medium was added with or without antioxidants. After 24 h, the medium was replaced without antioxidants and cells allowed to grow for a further 5 days. Cell colonies were counted and expressed as a percentage of untreated controls.

### Caspase inhibition

HSC-3 and HaCaT cells were seeded at 300 cells/well for clonogenic assay. Some cells were pre-incubated for 1 h with 50 μM Z-VAD-FMK caspase inhibitor. Cells were then incubated for 3 h with either 1 μg/ml AuNPs, or with 10 μM Antimycin A to induce apoptosis. Cells were then washed and allowed to grow for 6 days. Cell colonies were counted and expressed as a percentage of untreated controls.

### Cell-free hydroxyl radical assay

X-ray induced hydroxyl radical production was determined by the hydroxylation of coumarin-3-carboxylic acid (3-CCA) to fluorescent 7-hydroxycoumarin-3-carboxylic acid (7-OHCCA) [21,22]. A 5 mM stock solution of 3-CCA was prepared in 25 mM borate buffer of pH 9. Fifty μl aliquots of water, or 12 μg/ml AuNPs in water, were added to 50 μl 3-CCA in 96-well 3K molecular weight cutoff ultrafiltration plates (Acroprep Advance, Pall Corporation). Plates were irradiated with either 10 Gy of 6 MV X-rays at a dose rate of 5 Gy/min using a clinical linear accelerator (Versa HD, Elekta) at GenesisCare, Milton Keynes; or 10 Gy of 220 kV X-rays at a dose rate of 0.54 Gy/min using an Xstrahl 200 clinical orthovoltage system at Northampton Hospital. Control plates were not irradiated. Then, 100 μl ethanol was added per well to aid 7-OHCCA solubility; each plate was placed onto an empty receiving plate, and the nanoparticle-free filtrate was collected by centrifugation at 1500 x *g*. Quantification of 7-OHCCA fluorescence was performed with a FluoStar Optima plate reader (BMG Labtech). Excitation wavelength was set to 390 nm and maximum emission was detected at 450 nm [21].

### Cell irradiations

Cells were seeded in 24 well culture plates at between 300–1800 cells/well and allowed to adhere overnight. Cells were incubated with AuNPs for 3 h at the cancer cell IC50 (HSC-3 IC50 for skin cells; MCF-7 IC50 for breast cells). Cultures were then irradiated with 2–8 Gy of either 220 kV or 6 MV X-rays, as detailed in the previous section. The 6 MV dose was deposited at the level of the cell layer by beam shaping and Solid Water shielding (Gammex). Control plates were transported to each facility but were not irradiated. Cell colonies were counted after 6 days and expressed as the surviving fraction (SF).

SF data were normalised for chemotoxicity by multiplying the SF data for each type of AuNP by 1/SF at 0 Gy. A linear-quadratic function was fitted to the normalized SF versus dose (D) data, of the form SF = -exp(αD+βD^2^).

Radiation enhancement effects are reported as two measurements from normalized SF data. The Sensitivity Enhancement Ratio (SER_4Gy_) is a measure of cell senescence or death enhancement by AuNPs at 4 Gy, calculated as the ratio of SF without and with AuNPs [23]. The Dose Enhancement Factor (DEF_0.3_) is a measure of the gain in effective radiation dose due to AuNPs, calculated as the ratio of the dose with radiation only to dose with radiation and AuNPs at a SF of 0.3.

### Statistical analyses

Experiments were performed in triplicate. An unpaired t-test was used to compare two groups. To compare more than two groups, a one-way ANOVA was used with Bonferroni or Dunnett post-tests (GraphPad Prism 6.0 software).

## Results

### Physical characterisation of nanoparticles

The core size of the synthesised and citrate AuNPs was determined by TEM analysis, and demonstrated mean values between 1.7–2.4 nm, regardless of the ligands added during AuNP synthesis (Table 1, TEM images and histograms are shown in S1 Fig). Hydrodynamic diameters in water ranged from 4.5–6.6 nm, with no obvious link between ligand ratio and hydrodynamic size (Table 1). Except for the αGal-only AuNP and citrate AuNP, all of the AuNPs had a positive charge (Table 1). However, there was no obvious linear trend between zeta potential and sugar:PEG-amine ratio.

**Table 1:** AuNP physical characteristics.

Sugar:PEG-amine ratio	TEM diameter (nm)	DLS diameter (nm)	Zeta potential (mV)
αGal:PEG-amine 0:100	1.82 ± 0.99	5.08 ± 3.31	+43.0 ± 5.7
αGal:PEG-amine 40:60	2.03 ± 0.58	4.45 ± 2.35	+24.5 ± 7.2
αGal:PEG-amine 50:50	1.78 ± 0.57	6.29 ± 2.17	+45.4 ± 7.6
αGal:PEG-amine 60:40	1.65 ± 0.50	5.27 ± 2.31	+21.8 ± 5.7
αGal:PEG-amine 100:0	1.96 ± 1.00	5.13 ± 1.28	-16 ± 6.3
βGlc:PEG-amine 50:50	2.41 ± 0.81	6.15 ± 1.76	+24.1 ± 5.1
GlcNAc:PEG-amine 50:50	2.13 ± 1.09	6.11 ± 1.59	+30.9 ± 7.7
Citrate AuNP	2.04 ± 0.98	6.64 ± 2.17	-45.1 ± 13.9

AuNP diameter from TEM measurements (±SD), hydrodynamic diameter from DLS measurements in water (±SD), and zeta potential measured in 3.2% PBS pH 7.4 (±SD).

^1^H-NMR analysis of three different αGal:PEG-amine AuNPs revealed a slight preference for PEG-amine over αGal for attachment to AuNPs during synthesis. Thus, an input ratio of 75:25 αGal:PEG-amine yielded AuNPs with an actual ratio of 68:32. An input ratio of 50:50 αGal:PEG-amine yielded AuNPs with an actual ratio of 39:61. An input ratio of 25:75 αGal:PEG-amine, yielded AuNPs with an actual ratio of 17:83 (S2 Fig).

### AuNPs coated with sugar:PEG-amine are selectively toxic for skin cancer cells

**Fig 1:**
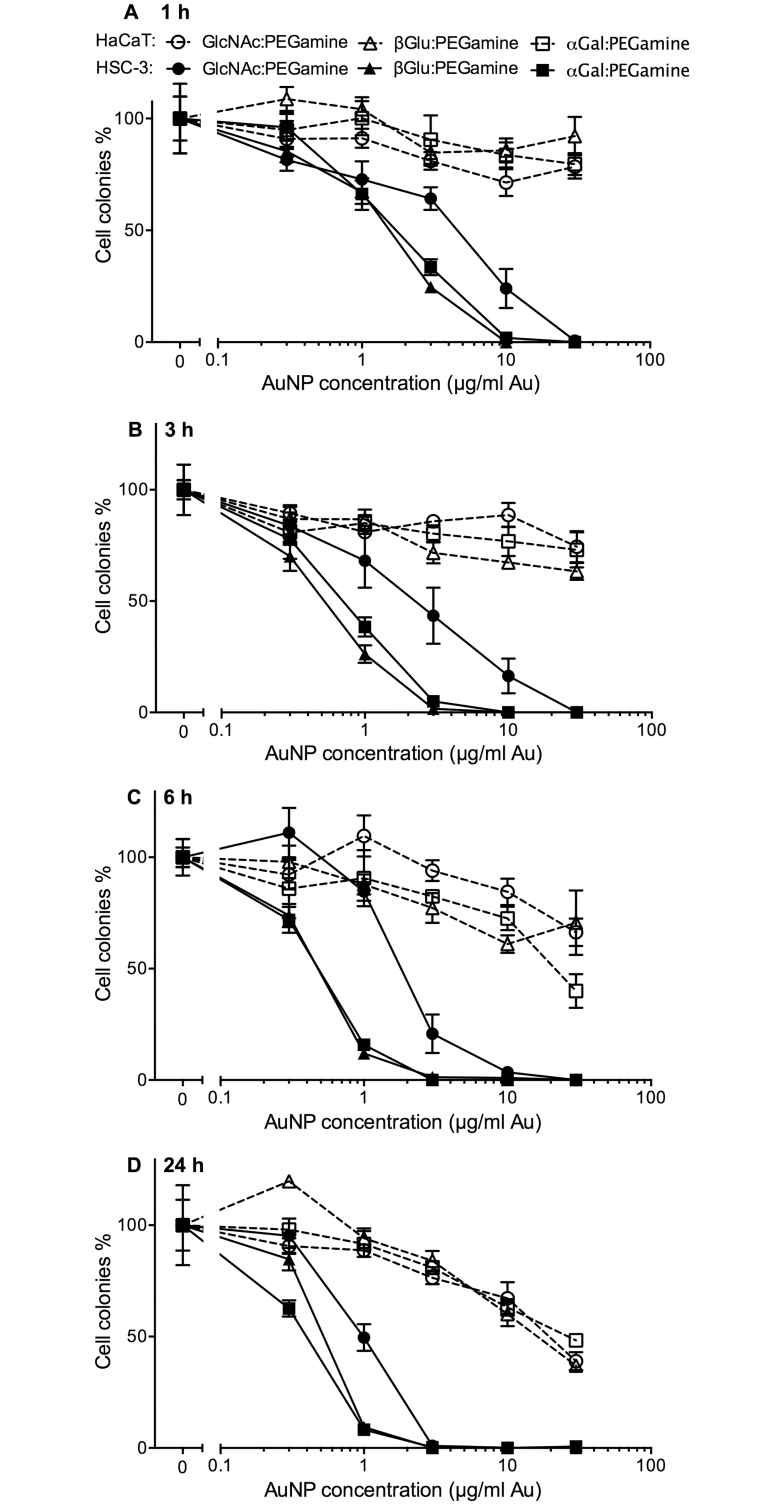
Clonogenic assay dose-response curves for three different 50:50 sugar:PEG-amine AuNPs on adherent HSC-3 and HaCaT cells. Cells were loaded with a range of AuNP concentrations for: A) 1 h, B) 3 h, C) 6 h and D) 24 h. The graphs represent the percentage of cell colonies compared to the no-nanoparticle control for each sugar:PEG-amine AuNP ±SEM.

Initially, three AuNPs, each bearing PEG-amine, but with different sugar ligands, were characterised for toxicity with HSC-3 and HaCaT skin cells. These were αGal:PEG-amine (50:50), βGlc:PEG-amine (50:50) and GlcNAc:PEG-amine (50:50). Cells were exposed to between 0.3–30 μg/ml of each nanoparticle for 1, 3, 6, or 24 h and then cell proliferation and survival assessed by clonogenic assay (Fig 1).

Three observations can be made from these data: 1) All AuNPs selectively compromise the proliferation and survival of HSC-3 cancer cells, compared with HaCaT normal keratinocytes; HSC-3 cell proliferation and survival show 2) a concentration dependent and 3) incubation time dependent decrease.

AuNPs bearing either αGal or βGlc sugars demonstrated similar toxicities for HSC-3 cells, while AuNPs bearing GlcNAc were around 3–5 times less toxic. Because Midatech Pharma have tested αGal functionalised nanoparticles in Phase I and II clinical trials, and therefore plenty of data exist on their stability and biocompatibility, the αGal ligand was selected for subsequent work, using a 3 h incubation time.

### The ratio of sugar:PEG-amine selectively affects cancer cell proliferation and survival

**Fig 2:**
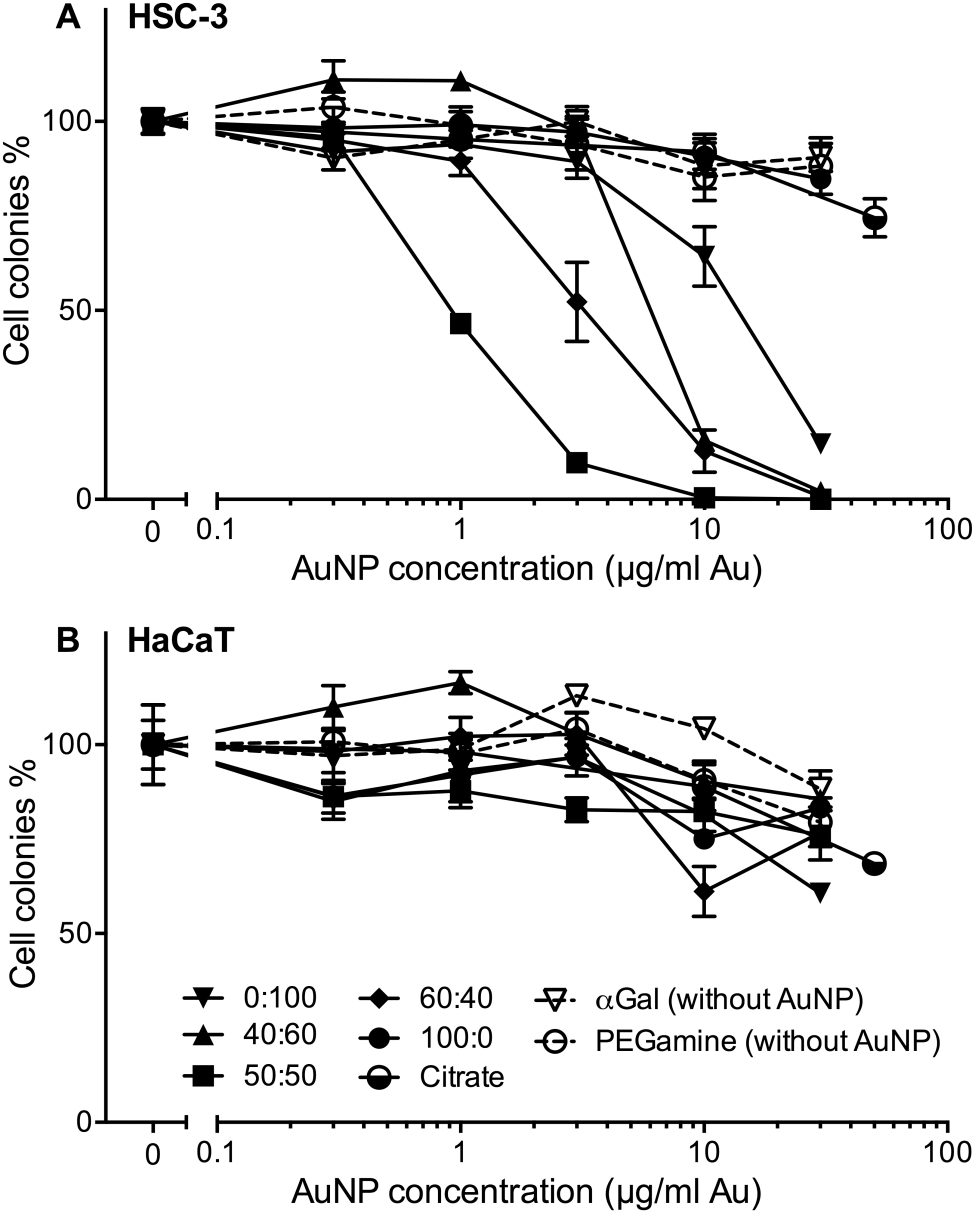
Clonogenic assay dose-response of different ratios of αGal:PEG-amine AuNPs, citrate-capped AuNPs, αGal only, or PEG-amine only, loaded for 3 h on adherent cells. a) HSC-3 cells, b) HaCaT cells. The graphs represent the percentage of cell colonies compared to the no-nanoparticle control ±SEM.

Having optimised the sugar ligand and loading time, the effect of different αGal:PEG-amine ratios on the toxicity of AuNP towards adherent HSC-3 cells and HaCaT cells, following 3 h exposure was investigated (Fig 2). Four different AuNP ratios demonstrated selective toxicity for HSC-3 cells (Fig 2A), while none of the AuNPs adversely affected HaCaT cell proliferation and survival up to 30 μg/ml gold content (Fig 2B). The greatest HSC-3 toxicities were observed respectively for: 50:50, 60:40, 40:60, and 0:100 αGal:PEG-amine ratios, whereas pure αGal AuNP (100:0) was not toxic. The αGal or PEG-amine ligands alone (without AuNP) were similarly not toxic. A citrate-capped AuNP was also not toxic. The HSC-3 IC50 values for the AuNPs, determined with cells loaded under adherent or suspension conditions, are presented in Table 2. Dose-toxicity graphs for suspension culture-loaded cells are presented in S3 Fig.

**Table 2:** Chemotoxicity of different AuNPs.

αGal:PEG-amine ratio	HSC-3 IC50 adherent (μg/ml Au)	HSC-3 IC50 suspension (μg/ml Au)
**100:0**	> 100	>100
**60:40**	4.2	1.8
**50:50**	0.8	3.4
**40:60**	6.8	6.2
**0:100**	13	17.4

Clonogenic assay IC50 values for HSC-3 cells exposed to different αGal:PEG-amine AuNPs for 3 h under adherent or suspension culture conditions.

### AuNP stability in culture medium

Four of the αGal:PEG-amine AuNPs were tested for their tendency to aggregate in DMEM culture medium plus 10% serum at AuNP concentrations of 10 μg/ml (a typical concentration that was used in cell culture). Of the four tested, the 0:100, 40:60 and 50:50 AuNPs did not demonstrate any aggregation, but the 100:0 AuNP did show aggregation (S4A–S4D Fig, respectively). In the absence of serum the AuNPs demonstrated pronounced aggregation (only shown for 50:50 AuNP in S4E Fig).

### AuNP cellular accumulation is not directly related to toxicity

**Fig 3:**
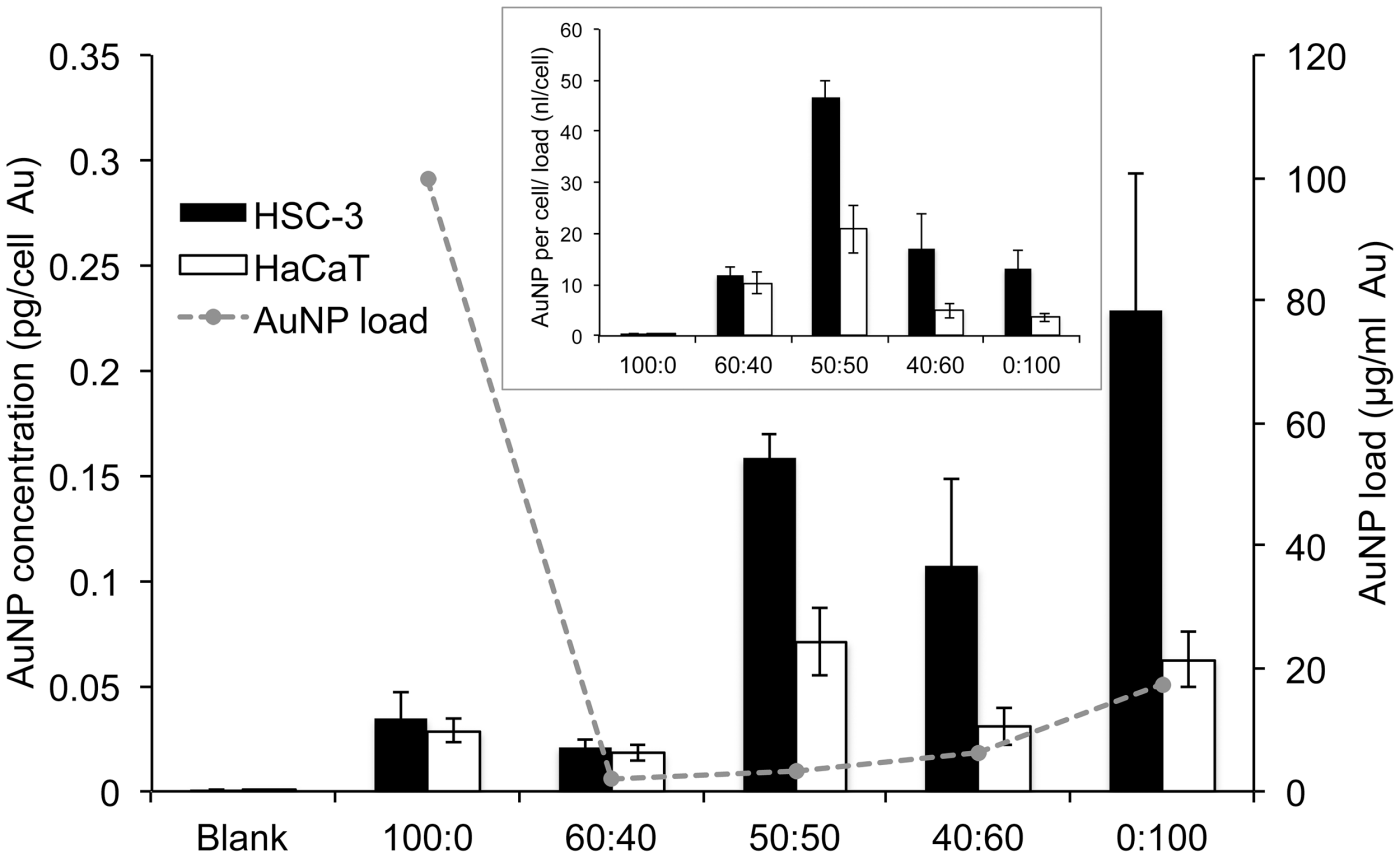
Amount of gold per cell (left axis) in HSC-3 cells and HaCaT cells loaded with the HSC-3 IC50 (suspension culture) concentrations of different αGal:PEG-amine AuNPs for 3 h. IC50 loading concentration (right axis) plotted as dotted line. Inset shows the same data re-plotted as gold per cell divided by AuNP loading concentration. All data are presented as mean value ± SEM.

To determine if there was a correlation between AuNP cellular accumulation and toxicity, cells were loaded with different ratios of αGal:PEG-amine AuNPs for 3 h at equitoxic doses corresponding to their HSC-3 IC50 concentrations under suspension culture conditions. Cellular accumulation was then quantified by ICP-MS. Despite being loaded at equitoxic concentrations, large differences were observed in the accumulation of the different αGal:PEG-amine AuNPs in HSC-3 cells after 3 h (Fig 3). For instance, the αGal-only AuNP was loaded at the highest concentration in HSC-3 cells (100 μg/ml) and yet showed the lowest accumulation (0.03 pg/cell). Conversely, the PEG-amine-only (0:100) AuNP and 50:50 AuNP demonstrated the highest accumulation in HSC-3 cells (0.23 pg per cell and 0.16 pg per cell), and yet this was achieved at loading concentrations of only 17.4 μg/ml and 3.4 μg/ml, respectively.

Re-plotting the data to normalise for the amount of AuNP loaded, revealed a trend in which AuNP accumulation per cell was highest with 50:50 αGal:PEG-amine and gradually decreased either side of this maximum as the αGal:PEG-amine ratio increased or decreased (Fig 3 inset). Thus, although high AuNP accumulation may be directly related to toxicity with the 50:50 αGal:PEG-amine AuNP, the relationship between uptake and toxicity for the other αGal:PEG-amine ratios does not appear to be so straightforward. For instance, the loading concentrations (equivalent to the IC50 values) are similar for the 60:40 and 50:50 αGal:PEG-amine AuNPs. However, the amount of AuNP accumulated per cell is markedly higher with 50:50 αGal:PEG-amine AuNPs, making this AuNP of interest for radiosensitisation studies.

### AuNPs accumulate in vesicles of cancer cells

**Fig 4:**
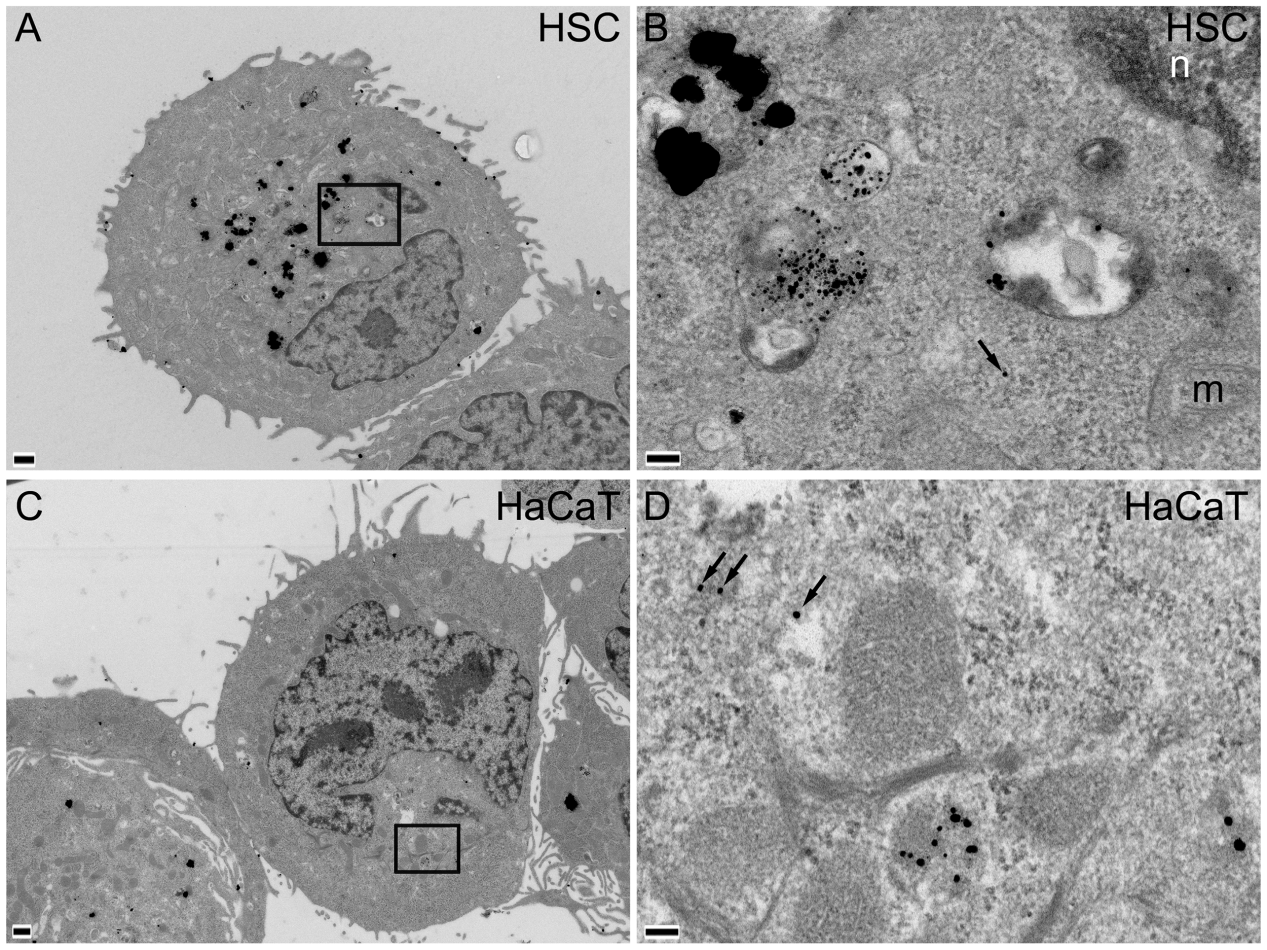
TEM images of A,B) HSC-3 and C,D) HaCaT cells incubated for 3 h with 10 μg/ml 50:50 αGal:PEG-amine AuNPs. Boxed areas in A and C are magnified in B and D, respectively. Arrows indicate AuNPs within cytoplasm; n, nucleus; m, mitochondrion; scale bars A,C are 500 nm; B,D are 100 nm.

**Fig 5:**
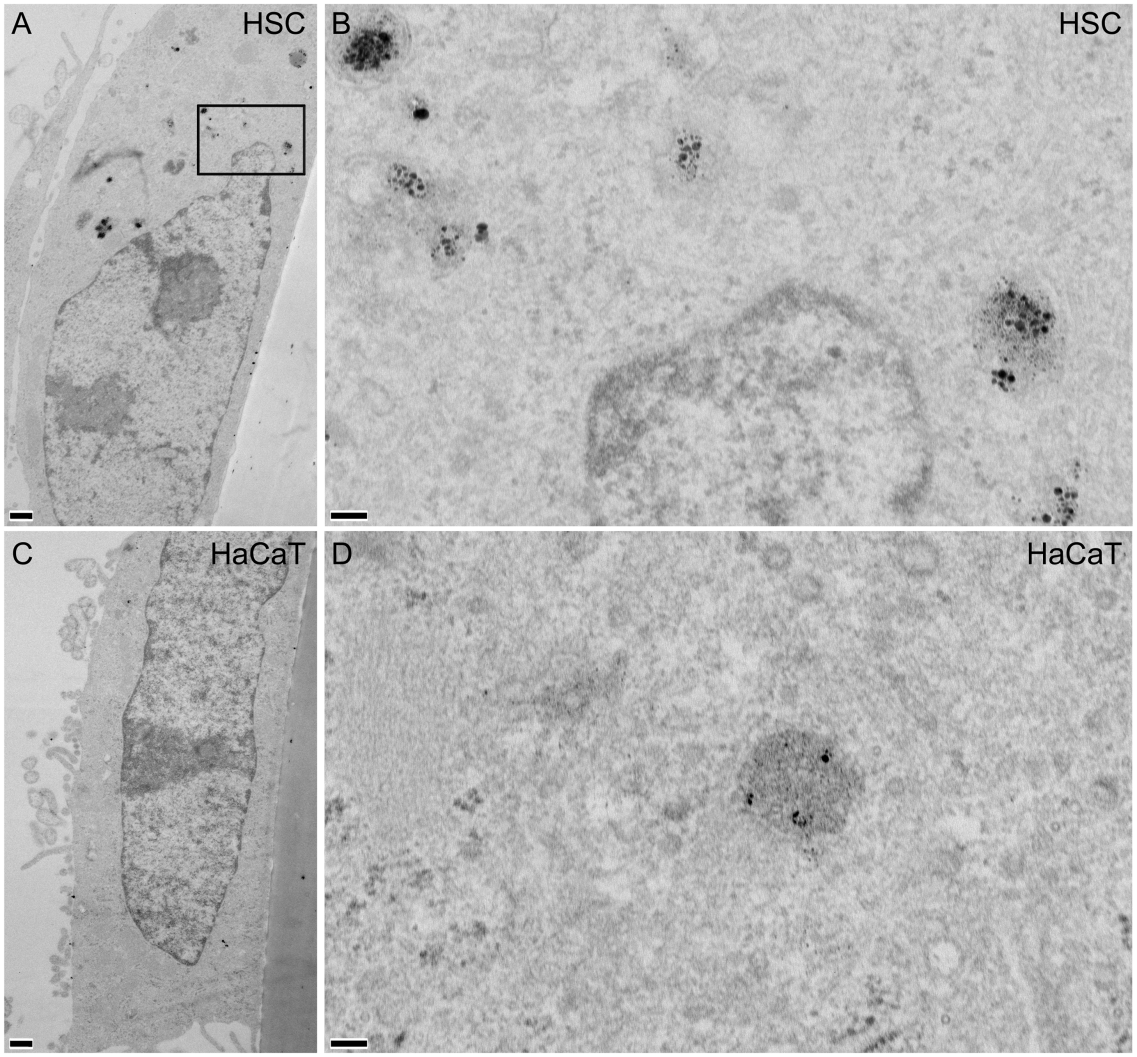
TEM images of A,B) HSC-3 and C,D) HaCaT cells incubated for 3 h with 10 μg/ml 0:100 αGal:PEG-amine AuNPs. Boxed area in A is shown magnified in B. Scale bars A,C are 500 nm; B,D are 100 nm.

TEM was used to reveal details of AuNP intracellular localisation (Figs 4 and 5). HSC-3 and HaCaT cells were incubated with 10 μg/ml 50:50 (Fig 4) or 0:100 αGal:PEG-amine AuNPs (Fig 5) for 3 h. AuNPs accumulated in juxtanuclear vesicles that resemble lysosomes, with strong preferential accumulation in HSC-3 cells, compared with HaCaT cells, consistent with ICP-MS data (Fig 3). Isolated AuNPs were occasionally found within the cytoplasm, but were never seen in mitochondria or the nucleus (Fig 4B and 4D).

### AuNP toxicity involves ROS and caspase-dependent cell death

**Fig 6:**
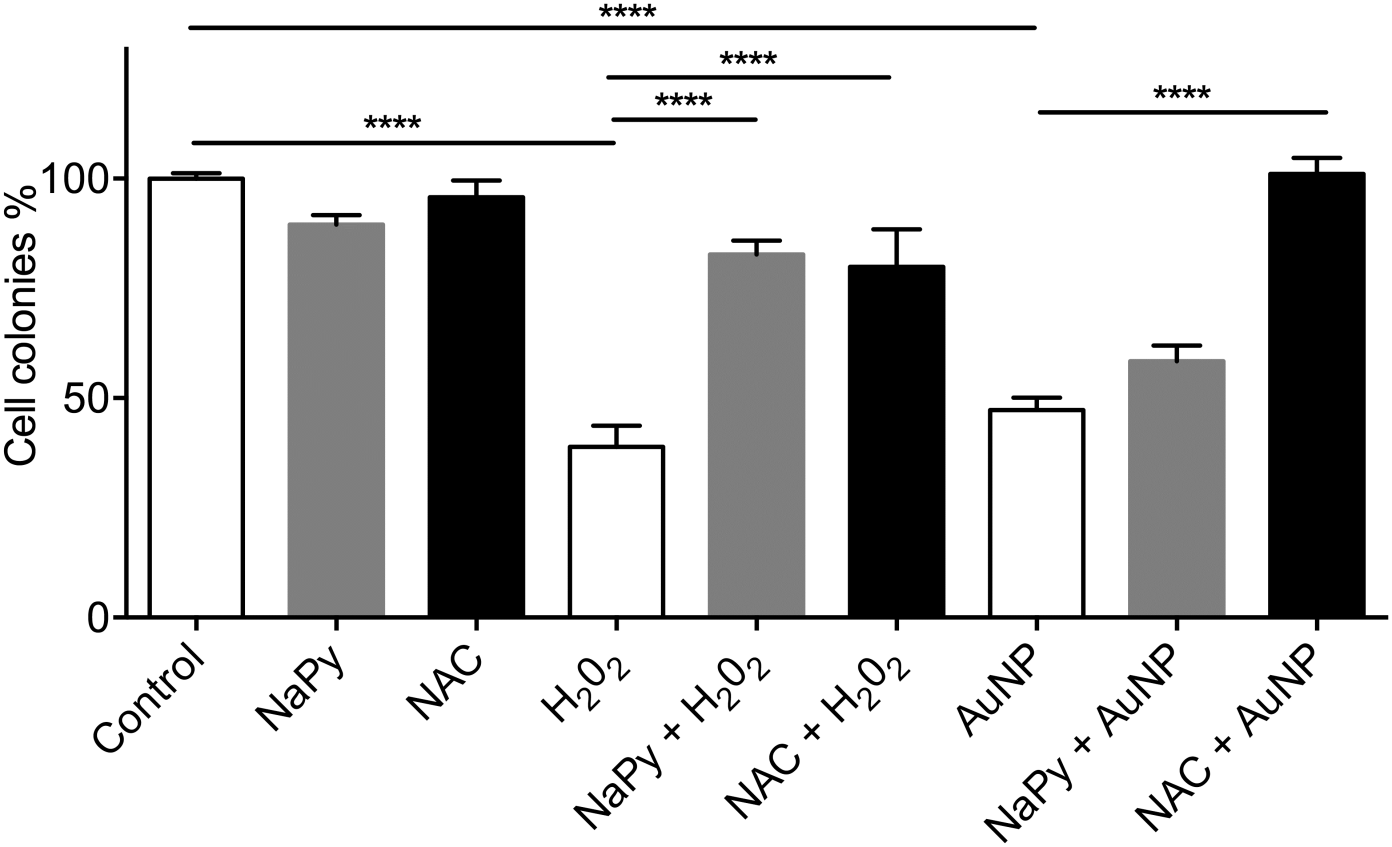
Clonogenic assay of HSC-3 cells exposed to 1 μg/ml 50:50 αGal:PEG-amine AuNPs in presence or in absence of 0.1 mM sodium pyruvate (NaPy) or 1 mM N-acetylcysteine (NAC) antioxidants. For each condition, n = 3 and data are presented ±SEM. **** Denotes a significant difference (P<0.0001 ANOVA, Tukey multiple comparisons post-test).

To determine whether ROS played a role in AuNP-mediated toxicity, HSC-3 cells were exposed to 1 μg/ml 50:50 αGal:PEG-amine AuNPs (chosen so as to be near the IC50 concentration) in the presence or absence of 0.1 mM sodium pyruvate or 1 mM N-acetyl cysteine antioxidants. N-acetylcysteine completely rescued the cells from AuNP-induced cell death, whilst sodium pyruvate gave a partial but not significant rescue of around 15% (Fig 6). Sodium pyruvate is known to scavenge extracellular ROS, but is poor at scavenging intracellular ROS [24], suggesting AuNPs being an intracellular source of ROS generation.

**Fig 7:**
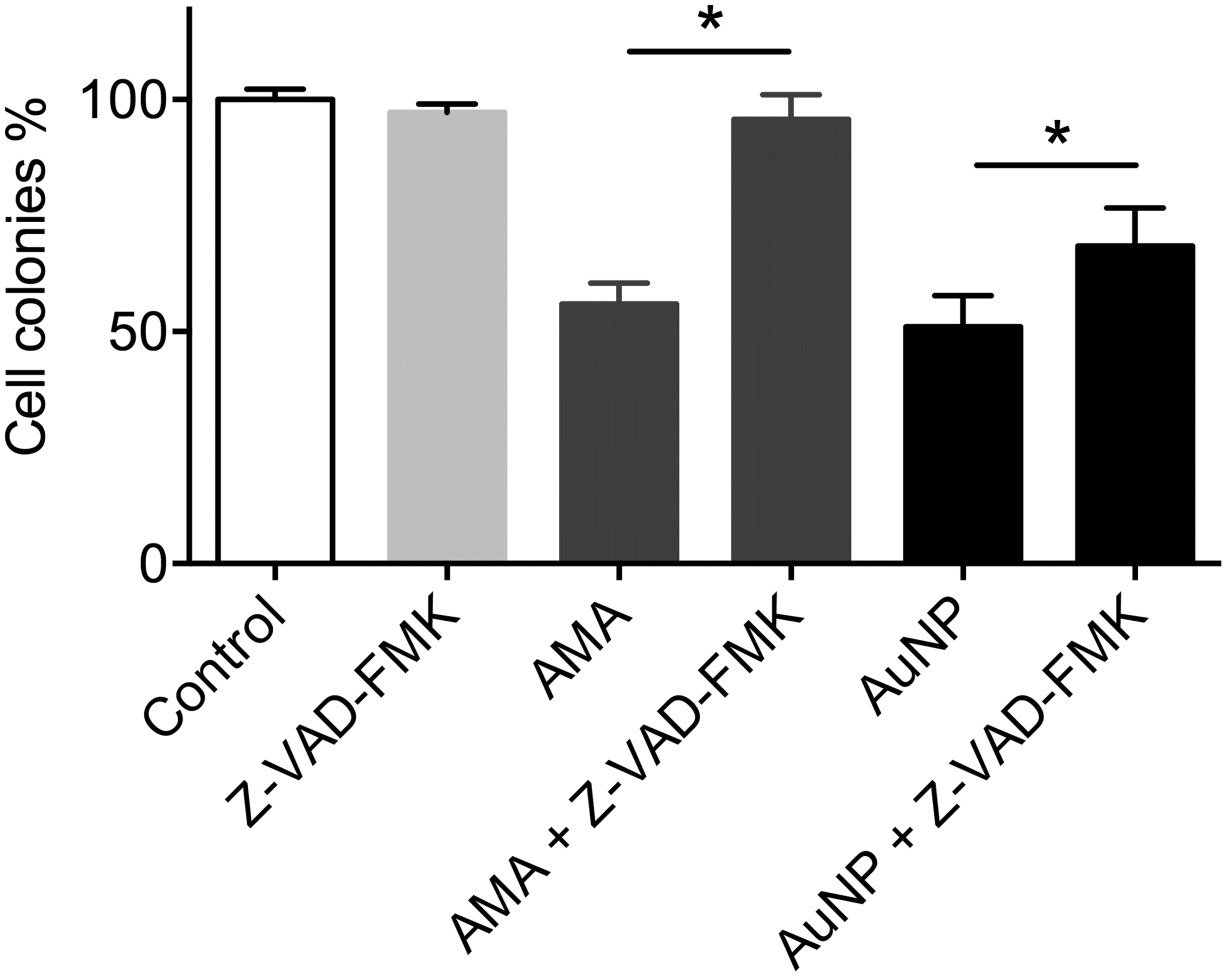
Clonogenic assay of HSC-3 cells, demonstrating a partial rescue of 50:50 αGal:PEG-amine AuNP-induced cell death by 50 μM Z-VAD-FMK caspase inhibitor. 10 μM Antimycin A was used as an apoptosis positive control. For each condition, n = 3 and data are presented ±SEM. * Denotes a significant difference (P<0.05 ANOVA, Tukey multiple comparisons post-test).

To determine whether apoptosis was involved in AuNP toxicity, cells were co-incubated with 1 μg/ml 50:50 αGal:PEG-amine AuNPs and the caspase 1/3 inhibitor Z-VAD-FMK (50 μM). Caspase inhibition resulted in a significant but incomplete rescue of AuNP-mediated cell death (Fig 7). By contrast, caspase inhibition gave a complete rescue of cell death using the apoptosis inducer, Antimycin A (10 μM) (Fig 7).

### Evaluation of intrinsic AuNPs radiosensitisation potential

The presence of a ligand shell on the AuNPs can interfere with irradiation-induced electron release from the gold core, and thus lower the intrinsic radiosensitisation potential [25,26].

**Fig 8:**
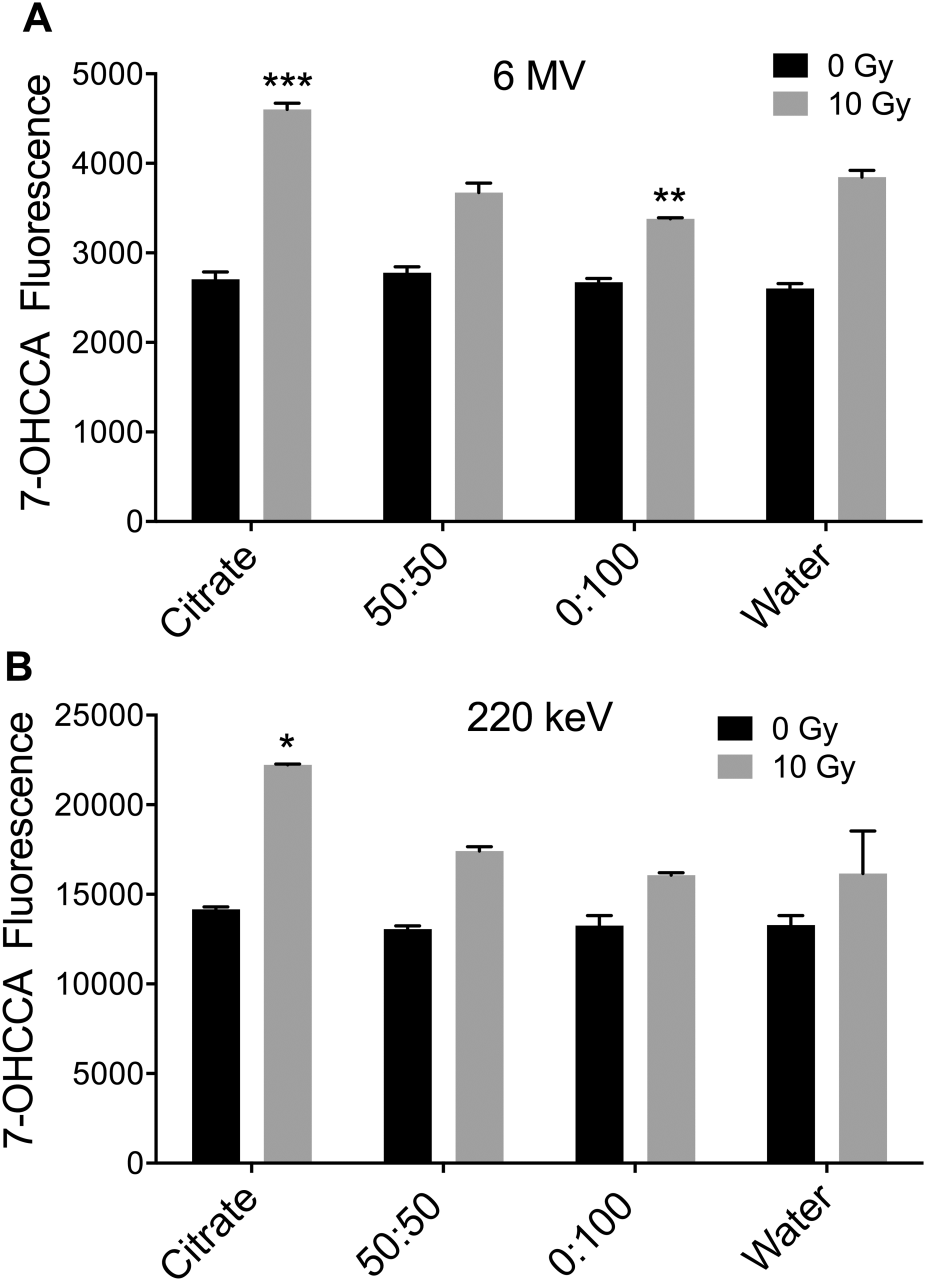
Hydroxyl radical formation assay of three different 6 μg/ml AuNP preparations in water with or without exposure to 10 Gy of A) 6 MV X-rays or B) 220 keV X-rays. Significant differences in fluorescence of 7-OHCCA probe following irradiation, compared to irradiated water only are indicated as *P*<0.05 *, *P*<0.01 **, *P*<0.001 *** (ANOVA with Dunnett’s multiple comparisons post test).

To investigate any such ligand shielding effect, a coumarin assay was used to examine irradiation-induced hydroxyl radical formation in cell-free aqueous solutions of AuNPs with different αGal:PEG-amine ligand shells, and citrate AuNPs that lack a thick ligand shell and therefore approach a ‘naked’ gold surface. A concentration of 6 μg/ml AuNPs was chosen for these experiments to maximize hydroxyl radical detection, since this was the highest AuNP concentration achievable with citrate-capped AuNPs (without resorting to filter concentration). Compared to water-only, 6 μg/ml citrate-capped 2 nm AuNPs (BBI) produced approximately 20% more 7-OHCCA fluorescence upon irradiation with 10 Gy of 6 MV X-rays. By contrast, radiation-induced 7-OHCCA production by 6 μg/ml 50:50 αGal:PEG-amine AuNPs was indistinguishable from that of water, while 6 μg/ml 0:100 αGal:PEG-amine AuNPs produced slightly less 7-OHCCA than water (Fig 8a). A similar trend was seen with 220 keV X-rays (Fig 8b). These data suggest that the 50:50 and 0:100 AuNPs would make poor radiosensitisers. However, ligand exchange by sulphur-containing proteins, such as glutathione, is known to occur on AuNPs [27], potentially allowing for sufficient reorganization of the αGal:PEG-amine ligand shell during intracellular processing to allow some radiosensitisation.

### Evaluation of AuNP biological radiosensitisation

**Fig 9:**
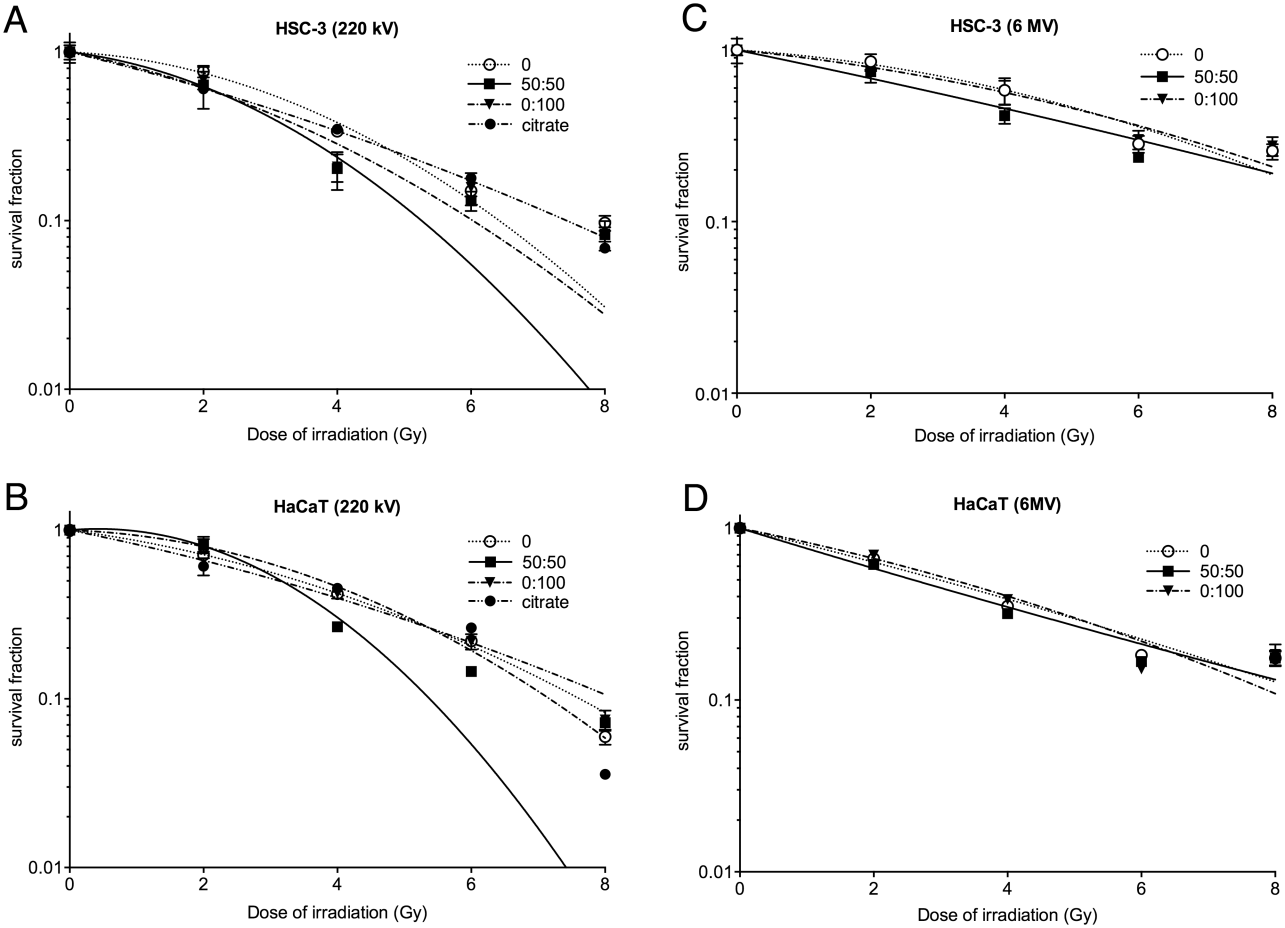
Normalised survival fractions of (A,C) HSC-3 and (B,D) HaCaT cells following exposure to αGal:PEG-amine AuNPs at their HSC-3 IC50 concentrations, followed by different doses of (A,B) 220 kV X-rays or (C.D) 6 MV X-rays. Data are presented as the mean survival fraction ±SEM. A linear-quadratic curve was fitted to each data series.

The 50:50 and 0:100 αGal:PEG-amine AuNPs demonstrated the highest cellular accumulation in HSC-3 cells (Fig 3) and were therefore selected for *in vitro* radiosensitisation experiments. HSC-3 and HaCaT cells were loaded with either 50:50 or 0:100 αGal:PEG-amine AuNPs at the HSC-3 IC50 for adherent cells (0.8 μg/ml for 50:50, 13 μg/ml for 0:100), or 1 μg/ml citrate-capped AuNPs (chosen to be near the HSC-3 IC50 concentration) for 3 h and then irradiated with doses of 2 to 8 Gy of either 220 kV X-rays or 6 MV X-rays. A linear-quadratic function was applied to the normalised SF data, which gave a good fit up to around 4 Gy, after which the slope deviated from linear-quadratic to become linear again (Fig 9). For this reason analyses of SEF were made at 4 Gy, while DEF was calculated at a survival fraction of 0.3 (DEF_0.3_), which in most cases also corresponded to around 4 Gy with AuNPs (Table 3). Radiosensitisation was increased in the presence of 50:50 αGal:PEG-amine AuNPs at both kV and MV energies (Fig 9, Table 3). However, the 0:100 αGal:PEG-amine AuNP was a poor radiosensitiser at kV energies and was slightly radioprotective at MV energies, compared to controls without AuNPs (Fig 9, Table 3), in keeping with the intrinsic radiosensitisation data (Fig 8).

**Table 3:** Sensitivity Enhancement Ratios and Dose Enhancement Factors calculated for skin cells.

Cell line (AuNP)	SER_4Gy_ 6 MV	SER_4Gy_ 220 kV	DEF_0.3_ 6 MV Dose 0/ Dose NP	DEF_0.3_ 220 kV Dose 0/ Dose NP
HSC-3 (50:50)	1.40	1.73	6.59/5.98 = 1.10	5.46/3.60 = 1.52
HSC-3 (0:100)	1.02	1.63	6.59/6.73 = 0.98	4.52/3.89 = 1.16
HSC-3 (citrate)	N/D	0.97	N/D	4.52/4.39 = 1.03
HaCaT (50:50)	1.10	1.08	4.97/4.22 = 1.18	5.61/4.02 = 1.40
HaCaT (0:100)	0.91	0.99	4.97/5.01 = 0.99	5.02/5.08 = 0.99
HaCaT (citrate)	N/D	0.93	N/D	5.02/4.95 = 1.01

### AuNPs are selectively chemotherapeutic to breast cancer cells

**Fig 10:**
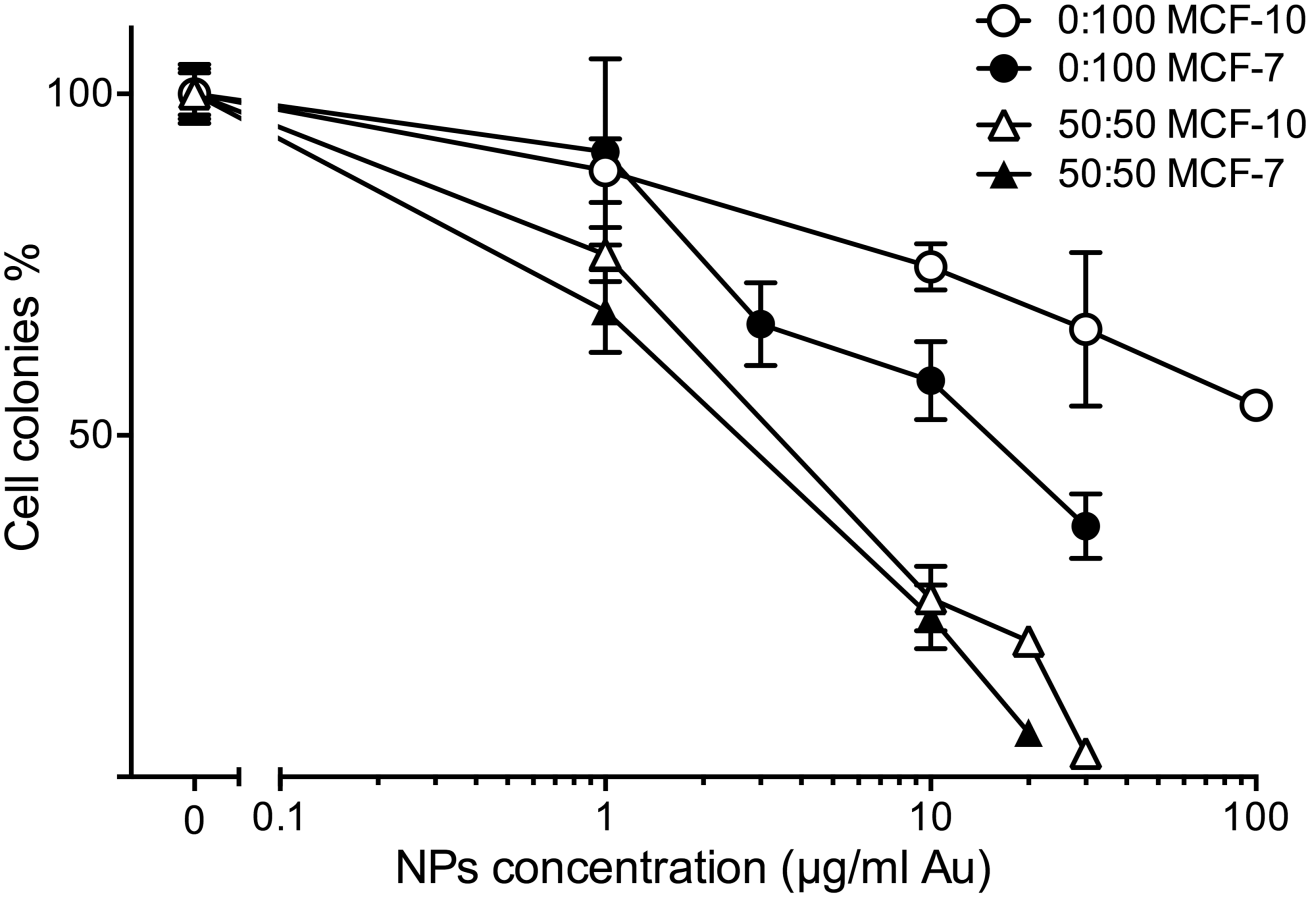
Clonogenic assay dose-response of adherent MCF-7 and MCF-10 cells exposed for 3 h to different concentrations of 50:50 or 0:100 αGal:PEG-amine AuNPs. The graphs represent the percentage of cell colonies compared to the no-nanoparticle control ±SEM.

Because 50:50 αGal:PEG-amine AuNPs demonstrated radiosensitisation with skin cancer cells, we investigated whether breast cells also demonstrated chemotoxicity and radiosensitistion with AuNPs. MCF-7 breast cancer cells and MCF-10 normal breast cells were loaded for 3 h with different concentrations of either 50:50 or 0:100 αGal:PEG-amine AuNPs under adherent conditions (Fig 10). For both AuNPs, toxicity was greater for MCF7 cells than with MCF-10 cells, although selectivity towards cancer cells was better with 0:100 αGal:PEG-amine AuNPs. The IC50 values for MCF-7 cells were 2.5 μg/ml for the 50:50 αGal:PEG-amine AuNP and 15 μg/ml for the 0:100 αGal:PEG-amine AuNP (Fig 10).

**Fig 11:**
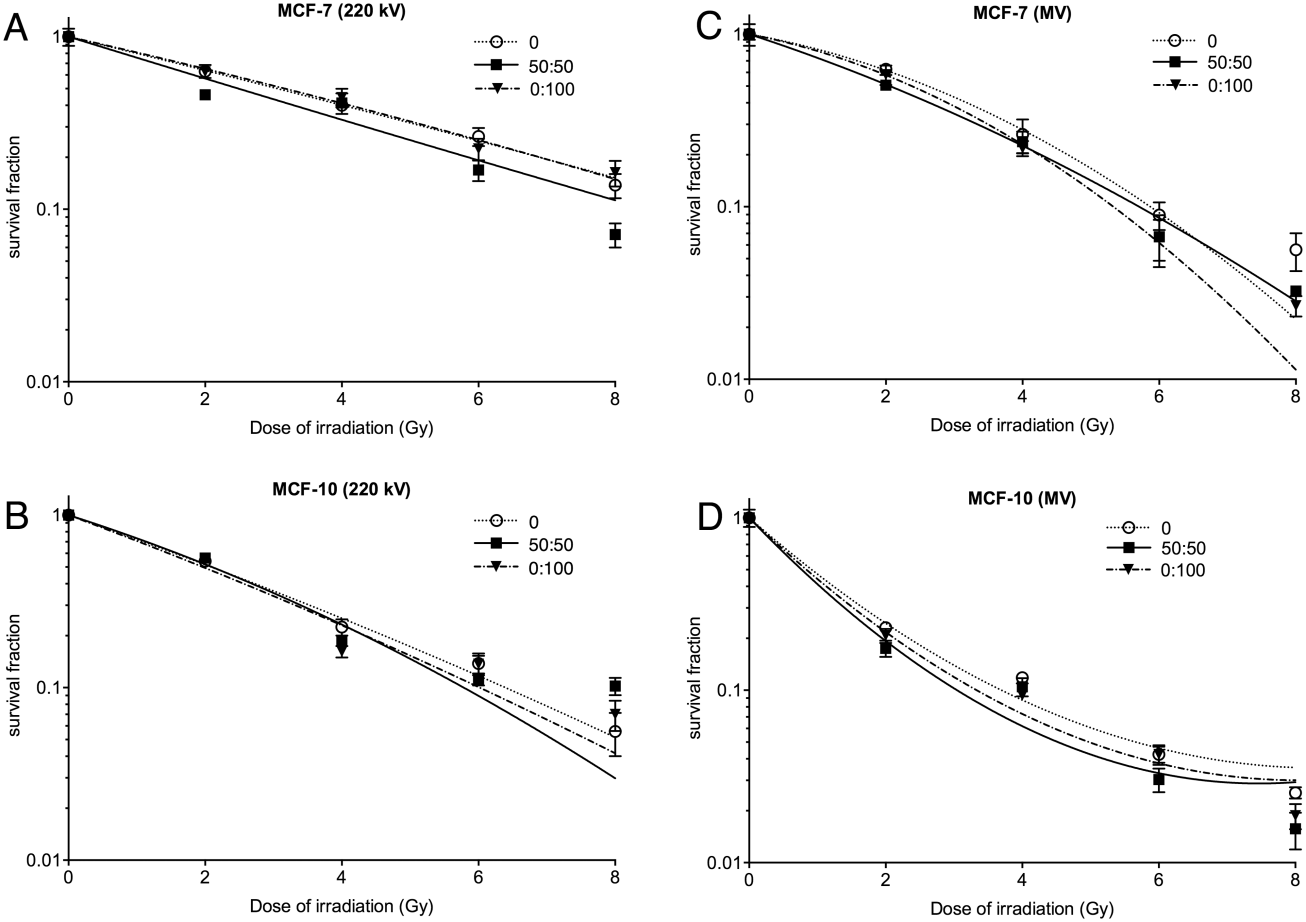
Clonogenic assay of (A,B) MCF-7 and (C,D) MCF-10 cells following exposure to αGal:PEG-amine AuNPs at their MCF-7 IC50 concentrations, then different doses of A,C) 220 kV X-rays or B,D) 6 MV X-rays.

To determine whether αGal:PEG-amine AuNPs radiosensitise breast cells, MCF-7 and MCF-10 cells were incubated for 3 h with the MCF-7 IC50 concentrations of αGal:PEG-amine AuNPs prior to irradiation with 2–8 Gy of either 220 keV X-rays or 6 MV X-rays. Clonogenic assay demonstrated radiosensitisation with 50:50 αGal:PEG-amine AuNPs, but negligible radiosensitisation with 0:100 αGal:PEG-amine AuNP (Fig 11, Table 4). Neither AuNP radiosensitised breast cells to the extent seen with skin cells.

**Table 4:** Sensitivity Enhancement Ratios and Dose Enhancement Factors calculated for breast cells.

Cell line (AuNP)	SER_4Gy_ 6 MV	SER_4Gy_ 220 kV	DEF_0.3_ 6 MV Dose 0/ Dose NP	DEF_0.3_ 220 kV Dose 0/ Dose NP
MCF7 (50:50)	1.11	0.97	3.85/3.34 = 1.15	5.22/4.35 = 1.20
MCF7 (0:100)	1.21	0.90	3.85/3.50 = 1.10	5.22/5.30 = 0.99
MCF10 (50:50)	1.12	1.20	1.67/1.40 = 1.20	3.53/3.40 = 1.04
MCF10 (0:100)	1.27	1.39	1.67/1.53 = 1.10	3.53/3.32 = 1.06

## Discussion

Current efforts to improve radiotherapy aim to either locally increase the radiation dose within the tumour or to magnify the effects of radiation damage by preventing cellular repair in tumour cells. From a practical viewpoint, an ideal treatment would combine both of these abilities in a single chemoradiosensitiser. Towards that goal, we find that AuNPs synthesised with specific ratios of sugar derivative and PEG-amine ligands are selectively toxic to skin cancer cells, with an IC50 of 0.8 μg/ml for the 50:50 αGal:PEG-amine AuNPs, and also act as radiosensitisers. Models calculate that a 1.96 nm AuNP contains 225 atoms [28], sitting between the measured values of 144 Au atoms for 1.68 nm AuNPs [29] and 333 Au atoms for 2.2 nm AuNPs [30]. Accordingly, we estimate that each 50:50 αGal:PEG-amine AuNP (mean diameter of 1.78 nm) contains around 180 Au atoms, making the HSC-3 IC50 concentration of 0.8 μg/ml gold equivalent to approximately 23 nM AuNP. These IC50 concentrations are the lowest we have found among published data for simple AuNPs that do not bear toxin ligands (ranging between 100 nM to 100 μM [12]). Importantly, we achieve these toxicities after only 3 h acute exposure to αGal:PEG-amine AuNPs, whereas most other studies report incubating cells with AuNPs for between 24–48 h [10,11,18,31–34].

Few studies have explored the selective toxicity of AuNPs for cancer cells. For instance, Patra et al [34] found selective chemotoxicity of 30 nm citrate-capped AuNPs towards A549 lung cancer cells after 48 h continuous exposure (IC50 100 nM), but no effect on HepG2 liver cancer cells. Butterworth et al [35] demonstrated a decreased proliferation rate following exposure of DU145 cells (but not MDA231-MB cells) to 10 μg/ml 1.9 nm AuroVist AuNPs. Exposure of these AuNP-loaded cells to 160 kV X-rays did not lead to appreciable radiosensitisation with DU145 cells (SER_2Gy_ 0.98), but did radiosensitise MDA231-MB cells (SER_2Gy_ 1.67), indicating their AuNPs were not dual chemoradiotherapeutic [35]. Changes in ligand organisation on AuNPs affect cell membrane interactions and thereby change the rate of cellular uptake [32,36]. We find that changes in the ratio of αGal:PEG-amine ligands dramatically affect the uptake and toxicity of AuNPs, with the highest uptake and toxicity seen with the 50:50 αGal:PEG-amine AuNPs.

Except for the 0:100 αGal:PEG-amine AuNPs, AuNP uptake per cell (inset in Fig 3) tends to be directly related to positive zeta potential (Table 1), although no relationship is apparent between uptake per cell and DLS diameter (Table 1). In serum-containing medium, the 0:100 and 50:50 αGal:PEG-amine AuNPs do not aggregate (S4 Fig) and remain as individual AuNPs when taken up into cells (Figs 4 and 5), except when they become highly concentrated within some lysosomes (Fig 4B). In contrast, 100:0 αGal:PEG-amine AuNPs do aggregate in serum-containing medium (S4E Fig), perhaps explaining their poor cellular uptake (Fig 3) and low toxicity (Table 2). Work is ongoing to unravel the mechanisms of selective cellular uptake and toxicity.

The mechanism of cell death with the 50:50 αGal:PEG-amine AuNPs involves elevated ROS and caspase-3 activation. This could imply interactions of AuNPs with mitochondria. However, AuNPs were not observed on or within mitochondria 3 h after AuNP loading, suggesting either an indirect or delayed interaction with these organelles. Several studies demonstrate ROS-mediated AuNP chemotoxicity, but this is generally seen in the continual presence of micromolar concentrations of AuNPs for 24 h [33,37].

Previous studies have demonstrated that adding a polyethylene glycol coating [38–40] or glucose coating to AuNPs [41,42] improves cellular uptake and radiosensitisation. It was reported that 15 nM glucose-coated AuNPs with 200 kV X-rays gave a 24 h SER_2Gy_ of 1.56 [41], while at 6 MV, 5 nM glucose-coated AuNPs gave a clonogenic SER_2.5Gy_ of 1.02 [42]. In both these cases, AuNPs were incubated with cells for 24 h. In a similar approach to ours, Zhu [43] compared the cytotoxicity and radiosensitisation of 20–30 nm AuNPs co-functionalized with galactose and polyethylene glycol, to citrate-capped AuNPs. They found no difference in chemotoxicity between these AuNPs with HepG2 cells following 24 h exposure (IC50 5 μg/ml). However, their galactose-polyethylene glycol-AuNPs demonstrated a better radiosensitisation than citrate AuNPs (DEF_0.37_ of 1.95 versus 1.46). In contrast, we find that with HSC-3 cells, 50:50 αGal:PEG-amine AuNPs are more chemotoxic than citrate AuNPs (IC50 0.8 μg/ml versus >50 μg/ml) and demonstrate better radiosensitisation (DEF_0.3_ 1.52 versus 1.03). Dense ligand coatings on AuNPs decrease irradiation-induced radical formation in cell-free assays [25,26], leading to the conclusion that improved radiosensitisation is likely due to a sufficiently increased cellular uptake of AuNPs, that outweighs their lower intrinsic irradiation-induced radical formation ability. We had hoped that by using short, hexameric, ethylene glycol chains, we could largely circumvent the ligand-dependent decrease in intrinsic radiosensitisation ability. However, we found that even these short ligand chains render the AuNPs no better than water in cell-free radiation-induced radical formation assays. Nevertheless, the observation of radiosensitisation in cells suggests that the ligand shell may become sufficiently modified *in vitro* to allow a proportion of secondary electrons to escape the nanoparticle surface and take part in radical production. This, in combination with the high chemotoxicity of these AuNPs and ease of synthesis, make them an excellent starting point for the rational design of further improvements to this novel chemoradiosensitiser platform.

## Supporting information

S1 Fig Size distribution of AuNPs. Size distribution histograms of AuNPs, measured from TEM images, plus representative TEM images for each AuNP. Scale bar is 20 nm.
(TIF)

S2 Fig Comparison of input and final AuNP αGal:PEG-amine ratios by ^1^H-NMR analysis. The input synthesis ratio and output actual ratio of αGal:PEG-amine were compared for three different AuNPs. A) a 75:25 αGal:PEG-amine mixture yielded an actual AuNP ratio of 68:32. B) a 50:50 αGal:PEG-amine mixture yielded an actual AuNP ratio of 39:61. C) a 25:75 αGal:PEG-amine mixture yielded an actual AuNP ratio of 17:83.
(TIF)

S3 Fig Chemotoxicity of AuNPs loaded in suspension culture. Clonogenic assay dose-response of different ratios of αGal:PEG-amine AuNPs loaded for 3 h under suspension culture conditions a) HSC-3 cells, b) HaCaT cells. The graphs represent the percentage of cell colonies compared to the no-nanoparticle control ±SEM.
(TIFF)

S4 Fig TEM images of AuNPs following incubation in culture medium at 10μg/ml. A-D) Different ratios of αGal:PEG-amine AuNPs were incubated for 3 h with DMEM culture medium containing 10% serum and were then imaged by TEM (scale bars are 20 nm). E) 50:50 αGal:PEG-amine AuNPs were incubated for 3 h with serum-free DMEM and were then imaged by TEM (scale bar is 2000 nm).
(TIF)

S1 Table Raw data. Excel spreadsheet containing all raw data.
(XLSX)
