# Supplementary figures and images for: Cancer-selective, single agent chemoradiosensitising gold nanoparticles
Source: PLoS One. 2017 Jul 10;12(7):e0181103. doi: 10.1371/journal.pone.0181103 (PMC5507319; doi:10.1371/journal.pone.0181103)

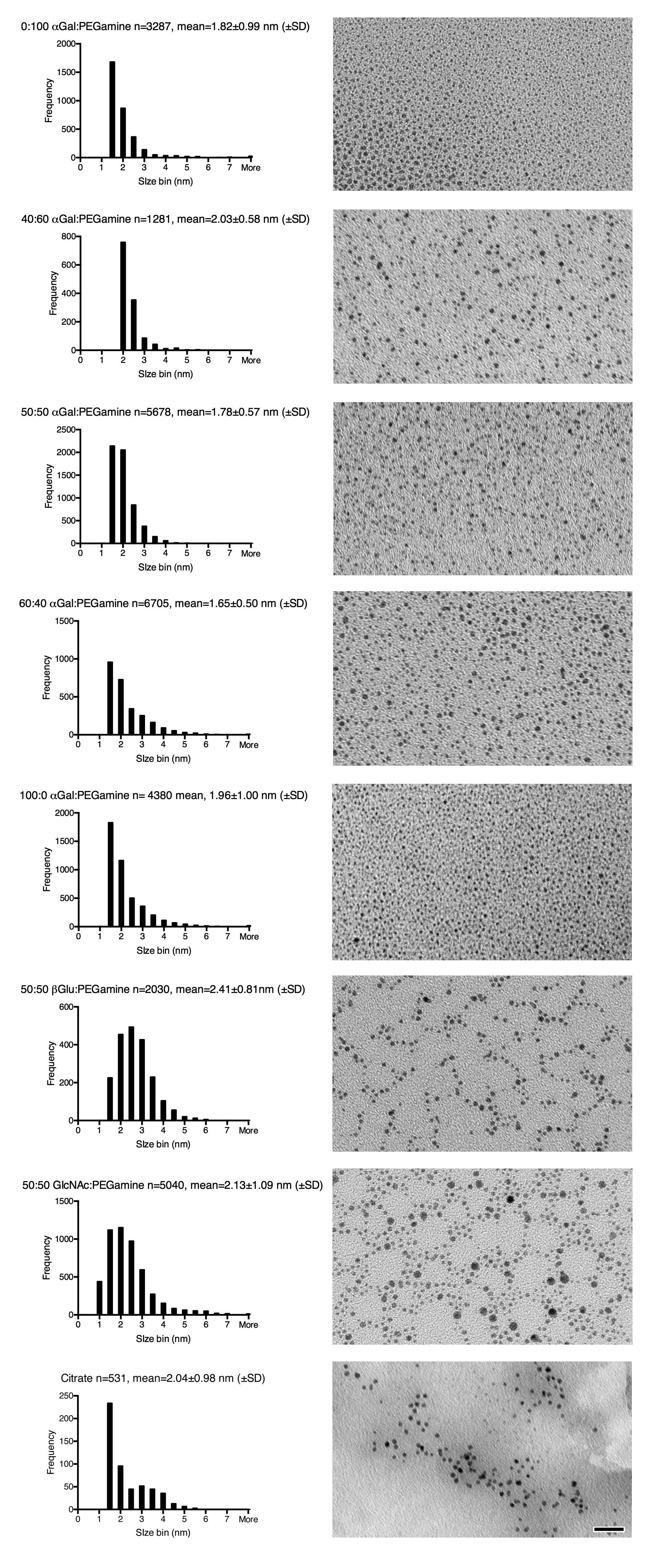

Supplement: S1 Fig — Size distribution histograms of AuNPs, measured from TEM images, plus representative TEM images for each AuNP. Scale bar is 20 nm. (TIF) [file pone.0181103.s001.tif]

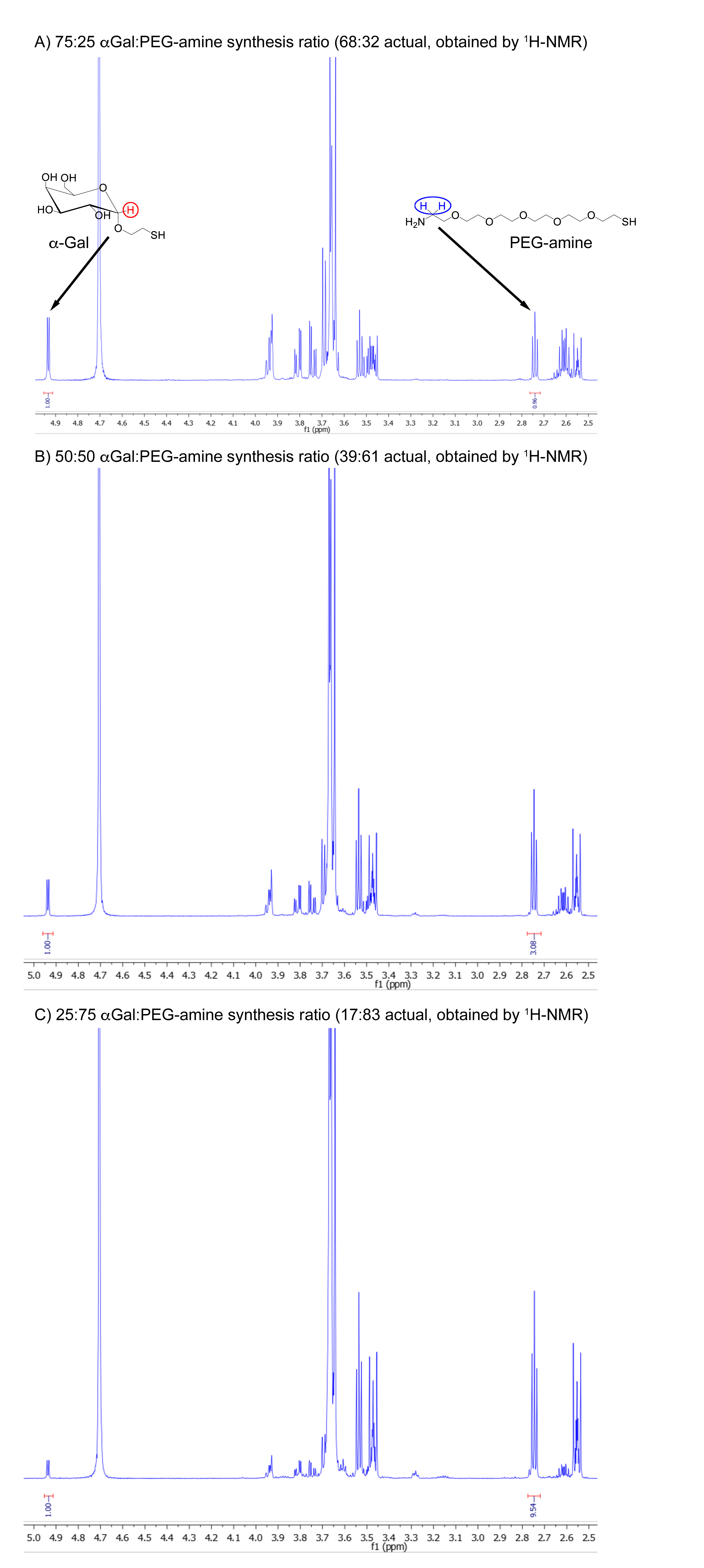

Supplement: S2 Fig — The input synthesis ratio and output actual ratio of αGal:PEG-amine were compared for three different AuNPs. A) a 75:25 αGal:PEG-amine mixture yielded an actual AuNP ratio of 68:32. B) a 50:50 αGal:PEG-amine mixture yielded an actual AuNP ratio of 39:61. C) a 25:75 αGal:PEG-amine mixture yielded an actual AuNP ratio of 17:83. (TIF) [file pone.0181103.s002.tif]

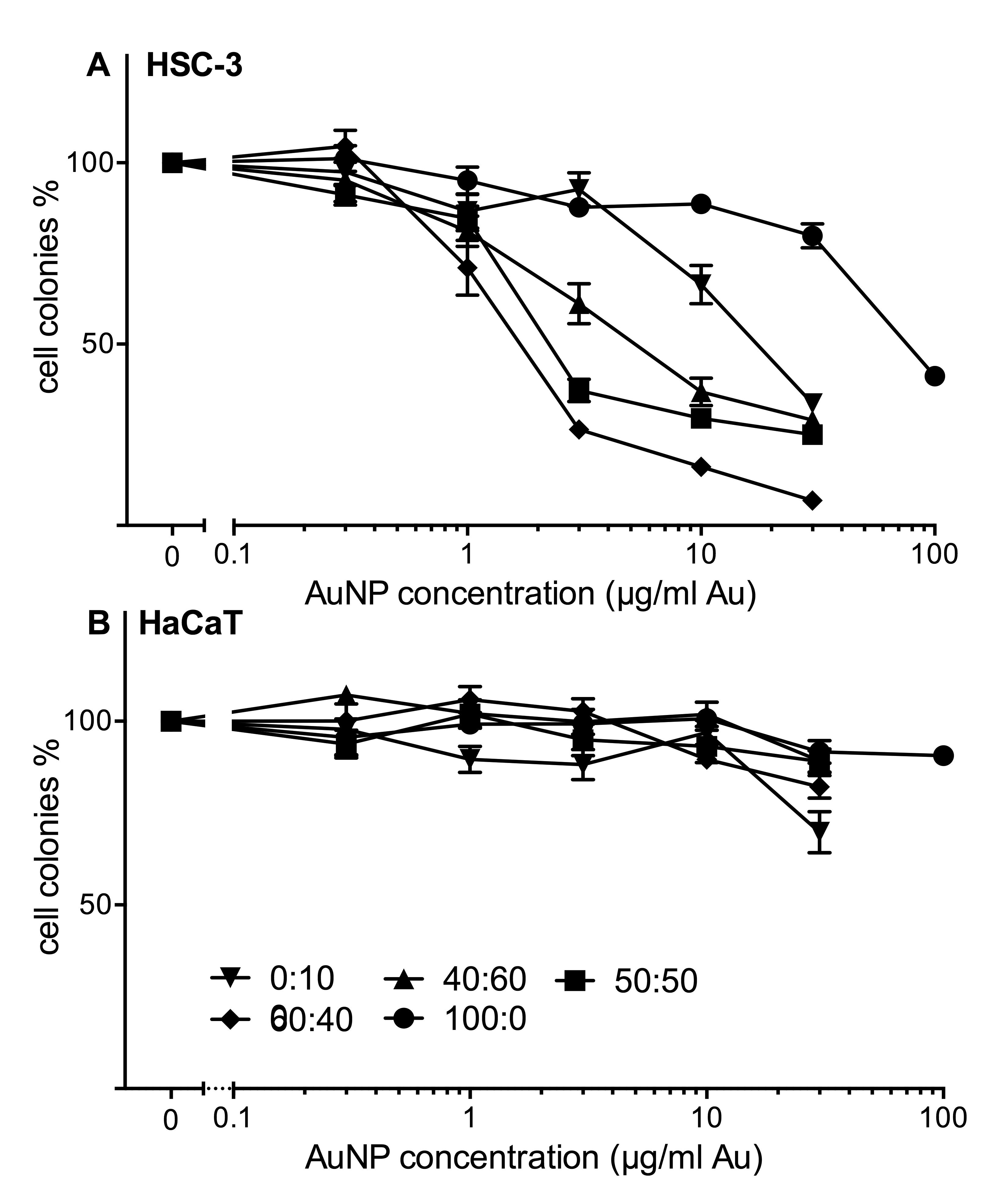

Supplement: S3 Fig — Clonogenic assay dose-response of different ratios of αGal:PEG-amine AuNPs loaded for 3 h under suspension culture conditions a) HSC-3 cells, b) HaCaT cells. The graphs represent the percentage of cell colonies compared to the no-nanoparticle control ±SEM. (TIFF) [file pone.0181103.s003.tiff]

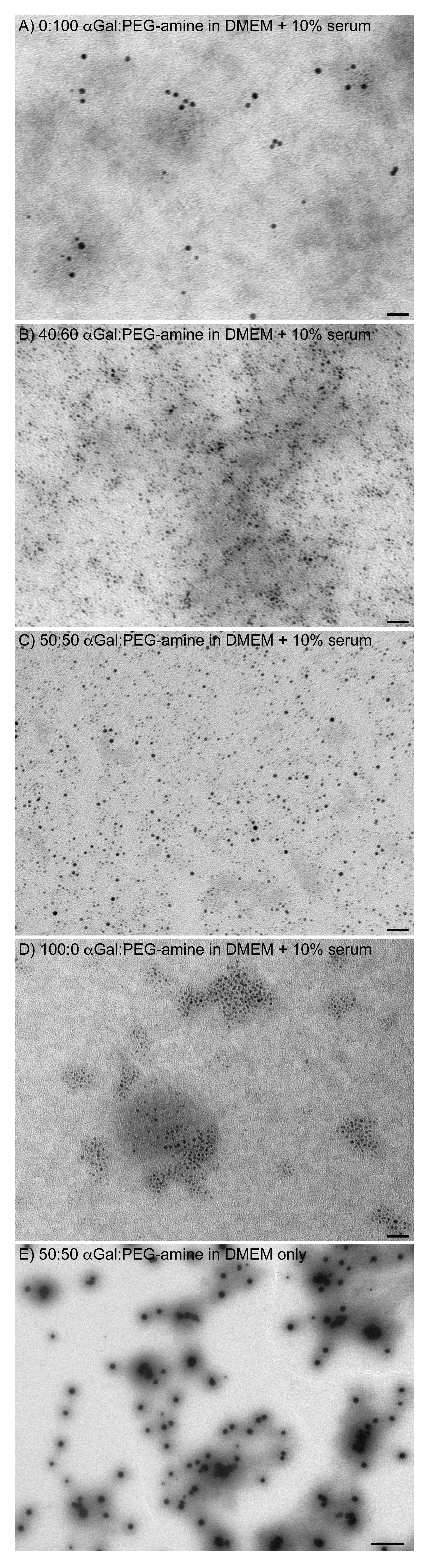

Supplement: S4 Fig — A-D) Different ratios of αGal:PEG-amine AuNPs were incubated for 3 h with DMEM culture medium containing 10% serum and were then imaged by TEM (scale bars are 20 nm). E) 50:50 αGal:PEG-amine AuNPs were incubated for 3 h with serum-free DMEM and were then imaged by TEM (scale bar is 2000 nm). (TIF) [file pone.0181103.s004.tif]
